# Deep learning in CRISPR-Cas systems: a review of recent studies

**DOI:** 10.3389/fbioe.2023.1226182

**Published:** 2023-07-03

**Authors:** Minhyeok Lee

**Affiliations:** School of Electrical and Electronics Engineering, Chung-Ang University, Seoul, Republic of Korea

**Keywords:** CRISPR-Cas system, CRISPR-Cas9, deep learning, guide RNA, genome editing, on-target activity, off-target activity, artificial intelligence

## Abstract

In genetic engineering, the revolutionary CRISPR-Cas system has proven to be a vital tool for precise genome editing. Simultaneously, the emergence and rapid evolution of deep learning methodologies has provided an impetus to the scientific exploration of genomic data. These concurrent advancements mandate regular investigation of the state-of-the-art, particularly given the pace of recent developments. This review focuses on the significant progress achieved during 2019–2023 in the utilization of deep learning for predicting guide RNA (gRNA) activity in the CRISPR-Cas system, a key element determining the effectiveness and specificity of genome editing procedures. In this paper, an analytical overview of contemporary research is provided, with emphasis placed on the amalgamation of artificial intelligence and genetic engineering. The importance of our review is underscored by the necessity to comprehend the rapidly evolving deep learning methodologies and their potential impact on the effectiveness of the CRISPR-Cas system. By analyzing recent literature, this review highlights the achievements and emerging trends in the integration of deep learning with the CRISPR-Cas systems, thus contributing to the future direction of this essential interdisciplinary research area.

## 1 Introduction

CRISPR-Cas systems, derived from a bacterial and archaeal adaptive immune mechanism, have rapidly evolved to become an indispensable tool in biotechnological and biomedical research for genome editing purposes (Wright et al., 2016). This revolutionary technique leverages the inherent capacity of these systems to target and modify DNA sequences with remarkable specificity and efficiency (Jinek et al., 2012).

The Clustered Regularly Interspaced Short Palindromic Repeats (CRISPR) and the CRISPR-associated proteins (Cas) form the two principal components of this system. The CRISPR locus in the bacterial genome contains short repetitive sequences interspaced by unique spacers derived from past invaders, such as viruses and plasmids (Barrangou et al., 2007). The transcription of this locus results in a precursor CRISPR RNA (pre-crRNA), which is processed into mature CRISPR RNAs (crRNAs). These crRNAs serve as guides for the Cas proteins to locate and cleave complementary sequences in the invading DNA, thereby providing adaptive immunity to the bacteria (Marraffini and Sontheimer, 2008).

CRISPR-Cas9, a Type II CRISPR-Cas system, has received substantial attention owing to its simplicity and adaptability for genome editing (Doudna and Charpentier, 2014). In this system, the crRNA is combined with a trans-activating crRNA (tracrRNA) to form a single-guide RNA (sgRNA). This sgRNA directs the Cas9 nuclease to the target DNA sequence, which is then cleaved by the nuclease, leading to insertions or deletions that can disrupt the targeted gene (Jinek et al., 2012).

An equally promising system is the CRISPR-Cas12, a Type V CRISPR-Cas system. Distinct from CRISPR-Cas9, CRISPR-Cas12 uses a single RNA molecule for both CRISPR array processing and target recognition, and cleaves DNA in a staggered pattern (Zetsche et al., 2015). This system offers unique advantages including targeting a wider range of sequences and providing collateral cleavage activity.

Despite the transformative potential of these CRISPR-Cas systems, accurate prediction of guide RNA (gRNA) activity remains a challenging task. This is where the recent advances in artificial intelligence (AI) methods, particularly deep learning, have begun to show their promise (LeCun et al., 2015). Deep learning models can capture complex patterns within data, making them ideally suited to predict gRNA activities based on sequence characteristics. They can, therefore, serve as an indispensable tool for accelerating the design and optimization process of gRNAs in CRISPR-Cas systems (Chen et al., 2019).

As deep learning rapidly evolves and the utilization of CRISPR-Cas systems continues to expand, it is crucial to continually revisit and evaluate the state of research in this area. Consequently, this review aims to investigate recent advances in deep learning models for predicting gRNA activity. Given the emerging importance of these technologies, this review will be instrumental in informing the ongoing development of accurate and efficient methods for gRNA prediction in CRISPR-Cas systems.

The expanding growth of the field of deep learning has underscored its potential in numerous applications, inclusive but not exclusive to the domain of genomics and genetic engineering (Eraslan et al., 2019). The rapid pace at which deep learning algorithms and methodologies are advancing necessitates continual scrutiny and updating of the state of knowledge in the field. The intertwining of deep learning with the CRISPR-Cas systems illuminates new horizons in the understanding and application of genetic engineering.

This review encapsulates the last 5 years of research, providing an exhaustive survey of deep learning’s current role in CRISPR-Cas systems. Given the swift growth of deep learning methodologies and the expanding CRISPR-Cas technology, this review is both relevant and indispensable. An examination of current literature reveals successes, challenges, emergent trends, and future possibilities in this domain. The review is expected to guide field researchers and practitioners, catalyzing further breakthroughs in the vibrant convergence of artificial intelligence and genetic engineering.

## 2 Literature analysis

### 2.1 Process of selecting papers

The purpose of this review is to present a comprehensive and focused synthesis of the most recent literature related to the use of deep learning methods in the CRISPR-Cas system. To achieve this, a systematic and diligent approach was adopted in the selection of papers.

The first stage of the paper selection process involved conducting a comprehensive search of the academic literature. The Web of Science (WOS) database, a widely recognized and extensive repository of scholarly literature, was primarily utilized for the purpose. Our search terms were carefully chosen to include essential keywords such as “deep learning”, “CRISPR-Cas”, and “neural network” to ensure the identification of relevant articles for our review.

This review exclusively incorporates articles published in peer-reviewed journals. This choice was determined by the stringent quality assurance processes that these publications undergo. Peer-reviewed articles are assessed by domain experts, thereby ensuring their scientific credibility. Moreover, they are acknowledged as significant platforms for disseminating scientifically rigorous and influential research.

While acknowledging the potential value of preprints and conference papers, a deliberate decision was made to solely focus on peer-reviewed journal articles. This selection criterion aims to uphold the dependability and validity of the review, safeguarding that the incorporated studies have undergone a meticulous vetting process. To maintain the freshness and innovation of this review, specific categories of articles, including review articles and perspectives, were purposefully omitted. This strategy underscores our intention to accentuate the integration of primary, research-based studies in alignment with the objectives of our review.

This review pertains exclusively to works published between 2019 and 2023, a timeframe judiciously chosen to ensure the study’s contemporaneity, pertinence, and inclusivity of recent innovations and patterns within deep learning applications for CRISPR-Cas systems. Noteworthy is the data collection for 2023, halted in May, to ensure the review captures the freshest insights and progressions in the domain. The quick advancement of CRISPR-Cas and deep learning technologies dramatically reshaped the landscape of research post-2019, making older studies nearly obsolete and consequently irrelevant for the present review. Given the spectacular progress in deep learning technology in recent years, prior research has been sidelined. Thus, focusing on the studies from 2019 to 2023 is judicious, rendering a meaningful and relevant critique of the state-of-the-art.

During the data collection process, additional information, such as the number of citations and publication details for each chosen article, was gathered. This data offered valuable insights into the scope, impact, and acceptance of the research within the scientific community. To provide a structured overview of the deep learning methodologies employed in the selected studies, the papers were categorized based on the specific objectives they pursued. This classification contributes to a comprehensive understanding of the deep learning landscape for the CRISPR-Cas system by enhancing comprehension of the various methodologies utilized. Table 1 provides a summary of the reviewed papers, with the majority of studies focused on predicting gRNA activities.

**TABLE 1:** Overview of Recent Studies Using Deep Learning in CRISPR-Cas system.

Research Topics	Brief description	Studies
Prediction of gRNA Activities	Uses deep learning methods to predict the efficiency or on- and off activities of guide RNAs (gRNAs) in the CRISPR/Cas system	Ameen et al. (2021), Shrawgi and Sisodia (2019), Dimauro et al. (2019), Xie et al. (2023), Xue et al. (2019), Wan and Jiang (2023), Zhang et al. (2020b), Li et al. (2022a), Xiang et al. (2021), Kirillov et al. (2022), Elkayam and Orenstein (2022), Niu et al. (2021b), Luo et al. (2019), Zhang et al. (2021a), Yang et al. (2023), Zhang et al. (2020c), Liu et al. (2020), Zhang and Jiang (2022), Vinodkumar et al. (2021), Zhang et al. (2020a), Vora et al. (2023), Lin et al. (2020), Charlier et al. (2021), Lin et al. (2022), Niu et al. (2021a), Jost et al. (2020), Xiao et al. (2021), Wang and Zhang (2019)
Prediction of CRISPR-Cas Editing Outcomes	Deep learning models to predict diverse outcomes of CRISPR-Cas editing, including mutational outcomes and cleavage efficiency	Li et al. (2021), Liu et al. (2022), Wang et al. (2021), Li et al. (2022b), Li et al. (2022c), Naert et al. (2020), Naert et al. (2021), Marquart et al. (2021), Zhang et al. (2021b)
Design of High-Activity gRNAs	Uses deep learning to design highly active gRNAs for CRISPR-mediated gene editing or epigenome editing	Baisya et al. (2022), Wang et al. (2019), Feng et al. (2021), Kim et al. (2020a)
Automated System Implementation	Using deep learning to automate specific processes in the application of CRISPR-Cas system	Cordero-Maldonado et al. (2019), Allen et al. (2022), Patino et al. (2021), Kanfer et al. (2021)
Nucleic Acid Detection	Utilizing CRISPR and deep learning for detection of nucleic acids related to specific diseases	Xie et al. (2022), Kang et al. (2022), Ding et al. (2023)
Anti-CRISPR Protein Identification	Utilizing deep learning to identify anti-CRISPR proteins	Wandera et al. (2022), Upmeier zu Belzen et al. (2019), Park et al. (2022)
Cas9 Variant Activity Prediction	Developing deep learning models to predict the activity and specificity of different Cas9 variants	Kim et al. (2020b)
Transcription Factor Binding Predictions	Using deep learning to predict transcription factor binding interactions	Avsec et al. (2021)
Analysis of Public Opinion	Employing deep learning to analyze public opinions about the CRISPR-Cas9 system	Muller et al. (2020)

### 2.2 Distribution analysis of publications in different journals

An analytical perspective on the dispersion of the chosen publications divulges insightful patterns and trends. The referenced articles manifest a broad distribution across various renowned journals, underscoring the global interest and engagement in deep learning for CRISPR-Cas systems. This dispersion across myriad scholarly avenues corroborates the cross-disciplinary appeal and reach of this research field.

A striking observation is the lack of monopolization by any single journal. The most represented journal accounts for a relatively small fraction, approximately 7.4%, of the total publications. This underlines the expansive and diversified publication landscape, where researchers are disseminating their findings across a multitude of platforms, potentially tailoring their target outlets based on their specialized readership and expertise.

Table 2 provides a statistical summary of the distribution of the selected papers across journals. The highest representation is seen in BMC Bioinformatics, Computational and Structural Biotechnology Journal, and Nature Communications, each containing four publications, accounting for around 7.4% of the total papers. Nucleic Acids Research and Bioinformatics follow suit with three papers each, contributing approximately 5.6% to the total publication count.

**TABLE 2:** Distribution of publications across journals.

Journal	Counts	Percentage (%)
Nature Communications	4	7.4
Computational and Structural Biotechnology Journal	4	7.4
BMC Bioinformatics	4	7.4
Nucleic Acids Research	3	5.6
Bioinformatics	3	5.6
Biomolecules	2	3.7
Scientific Reports	2	3.7
Biosensors and Bioelectronics	2	3.7
Nature Biotechnology	2	3.7
Misc	28	51.8
Total	54	100

Other significant contributors to the publication pool include Biomedicals, Scientific Reports, Nature Biotechnology, and Biosensors and Bioelectronics, each with two publications, constituting around 3.7% each of the total publication distribution. Besides, a multitude of other journals with a single publication, encapsulated under Misc., collectively accounts for 51.8% of the publications, emphasizing the extent and depth of research dissemination in this field. These findings signify that the research on deep learning for CRISPR-Cas systems transcends disciplinary boundaries, warranting publication in diverse scientific outlets ranging from bioinformatics and computational biology to genetics and molecular biology.

### 2.3 Quantitative analysis of the selected papers

In order to provide a more robust and statistically driven overview of the deep learning in CRISPR-Cas systems domain, a comprehensive evaluation of the selected academic articles is conducted. This analysis focuses on the distribution of citations and the temporal progression of publications, offering insights into the scholarly influence and the momentum of research in this field.

Beginning with citation statistics as a proxy for academic impact and community receptiveness, the median count is found to be seven. This value underscores a healthy level of interaction and endorsement of the considered studies. However, the average citation count, estimated at 16.1, exceeds the median, hinting at the existence of certain highly influential works that skew the mean upwards. This discrepancy between median and mean values reveals a distinct, multifaceted citation scenario among the analyzed papers.

**FIGURE 1:**
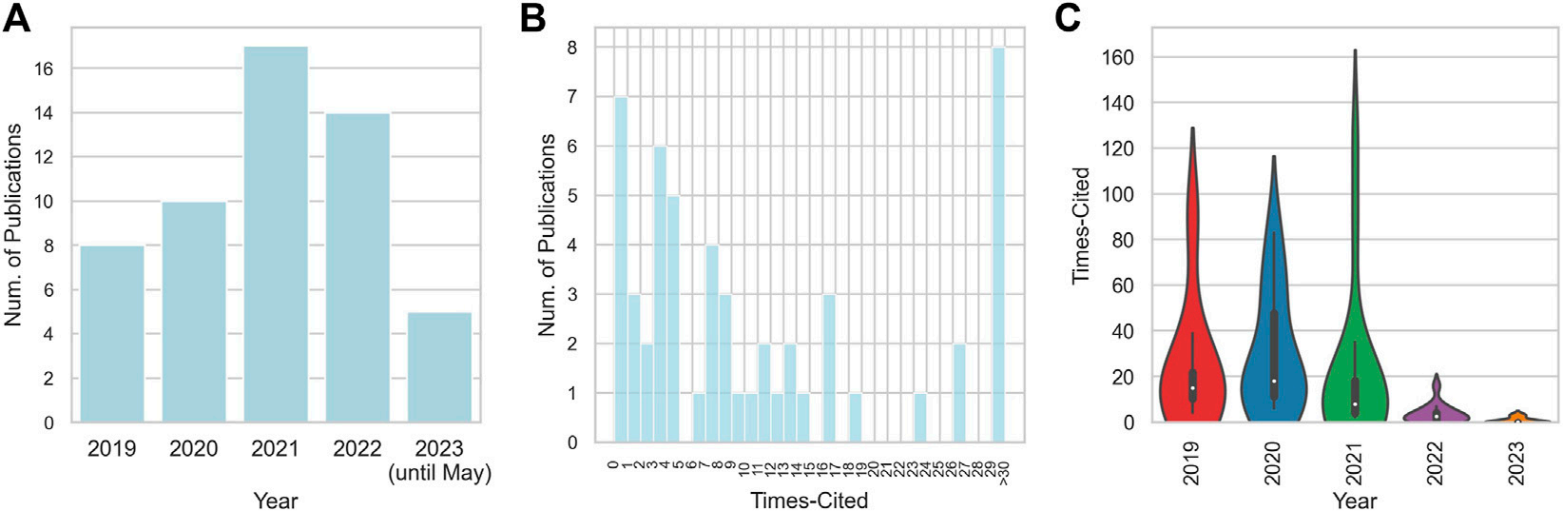
Overview of the distribution of publication years and citation frequencies. **(A)** Distribution of publication years; **(B)** Distribution of citation frequencies; **(C)** Violin plot for the relationship between citation frequencies and publication years.

Transitioning to a temporal inspection of the publications, an intriguing progression materializes. The year 2019 heralds the research in deep learning for CRISPR-Cas systems, with eight pioneering papers. This is followed by a modest ascension in 2020, where the count escalates to ten. Intriguingly, the year 2021 marks a critical juncture, featuring 17 publications, thereby contributing significantly to this emerging domain. For 2023, despite the incomplete data due to ongoing updates in WoS, five papers have already surfaced until April, suggesting a sustained trend of vigorous scholarly activity. A granular examination of the citation frequency and the year of publication for each paper is encapsulated in Figure 1.

This analytical exploration of the selected academic articles offers profound understanding of their scholarly impact and the trends shaping the field of deep learning for CRISPR-Cas systems. The analysis of the median and mean citation statistics illuminates the varied levels of scholarly reception, while the year-by-year publication count demonstrates the momentum of research output. Such insights contribute to an enriched understanding of the academic landscape and lay the groundwork for deeper exploration in this research arena.

## 3 Deep learning for prediction of gRNA activities

In CRISPR-Cas systems, a profound necessity exists to predict the activities of gRNAs with high precision and reliability. The spectrum of gRNA activities, spanning from on-target to off-target effects, lays the foundation for successful genome editing. However, the complexity of sequence-activity relationships inherent in gRNAs introduces a layer of intricacy, turning this task into a substantial challenge (Doench et al., 2016). Therefore, it is increasingly evident that conventional analytical approaches may be insufficient in tackling this issue, inviting more sophisticated computational methods to the forefront. The advent of deep learning has indeed provided an efficacious solution to this challenge.

Deep learning, a subset of artificial intelligence, involves neural networks with numerous layers (hence “deep”) that can model complex patterns in large datasets (LeCun et al., 2015). These neural networks learn from data by optimizing a loss function that captures the discrepancy between the predicted and true outcomes. They are capable of understanding hidden structures and relationships in data, making them particularly useful in predicting gRNA activities based on sequence characteristics.

The application of deep learning in predicting gRNA activities initially took form in studies focusing on the CRISPR-Cas9 system (Yin et al., 2019). These studies employed various types of deep learning architectures, including convolutional neural networks (CNNs) and recurrent neural networks (RNNs), to learn the intricate sequence-activity relationships of gRNAs. The learned models were capable of predicting on-target and off-target activities of gRNAs, thus guiding the selection of optimal gRNAs for specific genome editing tasks. The superior performance of these models underscored the potential of deep learning in this field.

Soon after, researchers began leveraging deep learning for the prediction of gRNA activities in the CRISPR-Cas12 system (Chen et al., 2018). CRISPR-Cas12, having unique properties compared to CRISPR-Cas9, posed different challenges in predicting gRNA activities. Nevertheless, deep learning proved its versatility by adapting to this system and delivering accurate predictions.

Recent advancements in the field have witnessed the emergence of more sophisticated deep learning models that offer greater predictive performance and interpretability. For instance, attention mechanisms, initially developed in the field of natural language processing, have been incorporated into deep learning models for gRNA activity prediction to provide insights into the importance of different nucleotides in a gRNA sequence. Such advancements are indicative of the growing maturity of deep learning applications in this field. Table 3 presents a compilation of recent investigations.

**TABLE 3:** Summary of the Contributions of Deep Learning Studies in Predicting gRNA Activities.

Author	Contributions
Ameen et al. (2021)	Proposed a hybrid CNN-SVR model for predicting gRNA activity in the CRISPR-Cas12 system, outperforming existing models
Shrawgi and Sisodia. (2019)	Developed a CNN model, DeepSgRNA, to predict the efficiency of sgRNAs in the CRISPR-Cas9 system
Dimauro et al. (2019)	Introduced CRISPRLearner, a CNN-based model for predicting sgRNA cleavage efficiency
Xie et al. (2023)	Proposed CRISPR-OTE, a CNN and biLSTM-based framework for gRNA on-target efficiency prediction
Xue et al. (2019)	Presented DeepCas9, a CNN-based model for identifying functional sgRNAs in the CRISPR-Cas9 system
Wan and Jiang. (2023)	Developed TransCrispr, a Transformer and CNN-based model for predicting sgRNA knockout efficacy
Zhang et al. (2020b)	Proposed a hybrid CNN-SVR system for predicting gRNA on-target efficacy in the CRISPR-Cas9 system
Li et al. (2022a)	Introduced a CNN and XGBoost-based model, CNN-XG, for predicting sgRNA on-target knockout efficacy
Xiang et al. (2021)	Created CRISPRon, a deep learning model for advanced gRNA efficiency predictions
Kirillov et al. (2022)	Presented a hybrid of Capsule Networks and Gaussian Processes for predicting gRNA cleavage efficiency
Elkayam and Orenstein. (2022)	Developed DeepCRISTL, a transfer learning model for predicting on-target editing efficiency in the CRISPR-Cas9 system
Niu et al. (2021b)	Introduced R-CRISPR, a deep learning model for predicting off-target activities in CRISPR-Cas9
Luo et al. (2019)	Presented DeepCpf1, a deep CNN model for predicting CRISPR-Cpf1 gRNAs on-target activity and off-target effects
Zhang et al. (2021a)	Proposed interpretable attention-based CNN models, CRISPR-ONT and CRISPR-OFFT, for predicting CRISPR-Cas9 sgRNA activities
Yang et al. (2023)	Developed EpiCas-DL, a deep learning framework for optimizing sgRNA design for CRISPR-mediated epigenome editing
Zhang et al. (2020c)	Developed DL-CRISPR, a deep learning model for predicting off-target activity in CRISPR-Cas9
Liu et al. (2020)	Introduced CnnCrispr, a model for predicting off-target propensity of sgRNA.
Zhang and Jiang. (2022)	Introduced CRISPR-IP, a CNN, BiLSTM, and attention layers-based model for CRISPR-Cas9
Charlier et al. (2021)	Introduced a novel encoding of sgRNA-DNA sequences to enhance deep learning off-target prediction in CRISPR-Cas9 gene editing
Lin et al. (2022)	Presented an AI approach integrating CNNs and attention module for quantifying CRISPR gene-editing off-target effects
Niu et al. (2021a)	Proposed an ensemble CNN model, sgRNACNN, for identifying high on-target activity of sgRNA in four agronomic species
Jost et al. (2020)	Used CRISPR interference to control gene expression, deriving rules governing mismatched sgRNA activity using deep learning
Xiao et al. (2021)	Proposed AttCRISPR, an interpretable model to predict sgRNA on-target activity, integrating encoding and embedding-based methods
Wang and Zhang. (2019)	Designed a CNN for predicting sgRNA activity in *E. coli*, emphasizing the importance of species-specific models in sgRNA prediction

In the CRISPR-Cas systems, several studies have proposed distinct methodologies using deep learning models to predict gRNA and sgRNA efficiencies and activities.

Studies utilizing hybrid models are evident in the field. For instance, Ameen et al. (2021) presented a hybrid CNN-SVR model for predicting gRNA activity within the CRISPR-Cas12 system. Further contributions include several studies proposing TransCrispr (Wan and Jiang, 2023) and a novel hybrid CNN-SVR system (Zhang et al., 2020b), respectively, for CRISPR-Cas9 system applications. Furthermore, hybrid models such as CNN-XG, a fusion of a convolutional neural network and XGBoost, were introduced (Li B. et al., 2022). Several other studies utilized CNN-based models, which developed DeepSgRNA (Shrawgi and Sisodia, 2019), CRISPRLearner (Dimauro et al., 2019), DeepCas9 (Xue et al., 2019), and CRISPRon (Xiang et al., 2021), respectively.

An alternative approach, combining multi-dimensional sequence information with prior knowledge, was adopted by Xie et al. (2023) in their proposition of CRISPR-OTE. Meanwhile, Kirillov et al. (2022) introduced a novel approach integrating capsule networks and Gaussian processes.

Studies have also proposed methods focused on predicting off-target activities, such as R-CRISPR (Niu R. et al., 2021) and DeepCpf1 (Luo et al., 2019). Other studies contributing to this segment include CnnCrispr (Liu et al., 2020), CRISPR-IP (Zhang and Jiang, 2022), and a graph convolutional network model (Vinodkumar et al., 2021), respectively.

Zhang G. et al. (2021) took a unique approach by proposing interpretable attention-based convolutional neural networks for predicting CRISPR-Cas9 sgRNA on-and off-target activities. Similarly, Vora et al. (2023) introduced a hybrid multi-task deep learning model, CRISP-RCNN, which emphasized the derivation of sequence features affecting Cas9 activity.

Models focusing on improving the predictability of off-target activities in CRISPR-Cas9 include CRISPR-Net (Lin et al., 2020) and novel encoding of sgRNA-DNA sequences (Charlier et al., 2021). Other studies such as focused on refining the quantification of off-target effects (Lin et al., 2022) and identifying high on-target activity in agronomic species (Niu M. et al., 2021), respectively.

Lastly, a few studies delved into controlling gene expression and improving predictive performance. For instance, Jost et al. (2020) utilized CRISPR interference to control gene expression, while Xiao et al. (2021) and Wang and Zhang (2019) proposed models that emphasized the importance of position-dependent nucleotide preferences and species-specific considerations, respectively.

### 3.1 Remarks on recent research trends

Recent advancements incorporate a variety of deep learning architectures, ranging from CNNs to RNNs, particularly those utilizing BiLSTM units, hybrid models, as well as attention mechanisms. This multiplicity of approaches signals the vibrancy and dynamism in the field, reaffirming the overarching view that no single method is universally optimal for every situation, thereby underscoring the need for bespoke solutions tailored to individual requirements.

A recurring trend is evident in the frequent utilization of CNNs. This adoption could be attributed to their superior capability to process sequence data, which is particularly pertinent in the context of gRNA sequences. CNNs are adept at identifying local patterns within sequences and are robust to variations in their position, which makes them particularly suitable for gRNA activity prediction.

The incorporation of BiLSTMs in numerous models elucidates another prevalent trend. The allure of BiLSTMs stems from their capacity to process sequences in both directions, capturing long-term dependencies in the sequence data. This characteristic makes them especially beneficial for tasks involving sequential data with complex dependencies, such as predicting the activities of gRNAs.

Interestingly, there seems to be an increasing interest in combining multiple learning models to form hybrid systems, frequently intertwining CNNs with other machine learning techniques such as SVMs or XGBoost. These hybrid models leverage the strengths of different methods, improving the robustness and performance of the predictive models.

Attention mechanisms, a recent innovation, also make their presence felt in the field. Derived from the domain of natural language processing, these mechanisms provide a mechanism to focus on crucial parts of the input sequence, enhancing the interpretability of the deep learning models.

Taken together, these trends reflect the swift evolution of deep learning methodologies in the domain of gRNA activity prediction. They indicate the field’s increasing sophistication and maturity while concurrently highlighting the plethora of opportunities that remain unexplored. These advancements, coupled with the constant evolution of deep learning architectures and methodologies, promise a future rich with possibilities for enhanced precision and efficiency in gRNA activity prediction.

## 4 Deep learning for prediction of CRISPR-Cas editing outcomes

The epoch of genomic engineering has been decidedly invigorated by the advent of the CRISPR-Cas9 system, an innovative tool that has dramatically transformed the landscape of genetic modification (Doudna and Charpentier, 2014). Despite its considerable potential, a lingering issue persists in the form of predicting the outcomes of CRISPR-Cas9 editing with a high degree of accuracy. The prediction of these outcomes is pivotal in genome editing, as it informs the design of guide RNAs (gRNAs) that direct Cas9 to target genomic sites (Gasiunas et al., 2012). This has prompted a profusion of studies employing deep learning methods to predict the on-target and off-target effects of gRNAs in the CRISPR-Cas9 system.

The allure of deep learning lies in its capacity to discern complex, non-linear relationships within data, surpassing the capabilities of traditional linear models (LeCun et al., 2015). Deep learning networks, composed of multiple interconnected layers, enable the transformation of the input data through a series of non-linear transformations to output a prediction. This process uncovers intricate patterns within the input data, a characteristic particularly pertinent in gRNA activity prediction given the complex nature of sequence-activity relationships.

The landscape of deep learning in predicting CRISPR-Cas9 editing outcomes is rapidly evolving, with growing interest in the incorporation of attention mechanisms to enhance interpretability. Originating from natural language processing, attention mechanisms direct the model to focus on pivotal parts of the input sequence, offering insights into the influence of different nucleotides in gRNA sequences (Vaswani et al., 2017). This innovation signifies the maturity of deep learning applications in this field, promising a future replete with possibilities for enhanced precision and efficiency in predicting CRISPR-Cas9 editing outcomes. A summary of contemporary research endeavors is depicted in Table 4.

**TABLE 4:** Summary of the contributions of deep learning studies in CRISPR-Cas9 editing outcomes.

Author	Contributions
Li et al. (2021)	Proposed an end-to-end deep learning framework named CROTON, an approach used deep multi-task convolutional neural networks and neural architecture search (NAS) to automate both feature and model engineering
Liu et al. (2022)	Developed Apindel, a deep learning model employing BiLSTM and Attention mechanism, to predict a comprehensive range of Cas9-generated mutational outcomes, surpassing previous models in accuracy
Wang et al. (2021)	Introduced EditPredict, a CNN-based model that accurately predicts RNA editing events in humans, including those resulting from CRISPR-Cas9 knockout of the ADAR1 enzyme
Li et al. (2022b)	Devised SeqGAN, a model combining CNN and an adversarial network, to predict CRISPR off-target cleavage sites, achieving superior performance in cross-validation tests
Li et al. (2022c)	Proposed machine learning approaches, including deep learning, for identifying human blood cells with CRISPR-mediated fetal chromatin domain (FCD) ablations, with promising results in the prediction of edited cells
Naert et al. (2020)	Applied predictive modeling of editing outcomes to maximize CRISPR-Cas9 phenotype penetrance in *Xenopus* and zebrafish embryos, improving phenotype penetrance in the F0 generation
Naert et al. (2021)	Introduced CRISPR-SID, a method using deep learning to predict double-strand break repair patterns for identifying genes essential for tumorigenesis in a *Xenopus* tropicalis desmoid tumor model
Marquart et al. (2021)	Developed BE-DICT, a deep learning model incorporating attention mechanisms, to predict base editing outcomes, providing a versatile tool for genome editing
Zhang et al. (2021b)	Conducted a comprehensive evaluation of the editing efficiency of several Cas9 variants and utilized a deep learning model to verify and predict SpRY off-target sites, informing the refinement of Cas9 variants for precise editing

In the prediction of CRISPR-Cas9 editing outcomes, multiple studies have been conducted, each offering unique methodologies and insights. For instance, the work presented by Liu et al. (2022) introduced the Apindel model, a framework incorporating BiLSTM and Attention mechanism. The model displayed enhanced performance in prediction detail and accuracy relative to its predecessors. Meanwhile, Marquart et al. (2021) established BE-DICT, a sophisticated model utilizing attention mechanisms, trained on a vast genetic sequence library, which showed a notable precision in predicting base editing outcomes.

The application of CNNs in this domain has also been substantial. Wang et al. (2021) offered EditPredict, a CNN model that has been shown to predict RNA editing events in humans with impressive accuracies. The model’s functionality extends to reference genome sequences and can account for single nucleotide variants in personal genomes. On a similar vein, Li W. et al. (2022) brought forth SeqGAN, a novel model amalgamating CNN and a sequence-generating adversarial network, yielding enhanced predictions for CRISPR off-target cleavage sites.

Machine learning approaches have also been utilized to identify specific biological changes in cells. For instance, Li Y. et al. (2022) proposed two unique models utilizing multilayer perceptron algorithms and deep learning to detect human blood cells with CRISPR-mediated fetal chromatin domain (FCD) ablations. The models displayed promising predictive abilities for genetically edited cells.

Exploring predictive modeling further, Naert et al. (2020) developed a neural network trained in mouse embryonic stem cell cultures to predict editing outcomes in *Xenopus* and zebrafish embryos. The model successfully predicted repair outcomes leading to a considerable increase in phenotype penetrance in the F0 generation.

A substantial contribution to the field of tumorigenesis research was made by Naert et al. (2021), who introduced CRISPR-SID. This approach integrated multiplexed CRISPR-Cas9 genome editing with deep learning, predicting double-strand break repair patterns to identify genes vital for tumorigenesis, suggesting a druggable potential of key genes.

Finally, the study by Zhang W. et al. (2021) exemplifies the successful combination of high-throughput sequencing and deep learning. They assessed the efficiency, specificity, and PAM compatibility of various Cas9 variants, using a deep learning model to validate and predict SpRY off-target sites, thereby informing the refinement of Cas9 variants for precise genome editing.

### 4.1 Remarks on recent research trends

From the comprehensive overview of recent studies as depicted in Table 4, several emergent trends shaping the intersection of deep learning and CRISPR-Cas9 editing outcomes prediction are discerned. One salient trend is the increasingly multifaceted nature of the adopted deep learning architectures, often manifesting in combinations of well-established neural network paradigms, such as the marriage of BiLSTM and Attention mechanisms or the fusion of CNNs and adversarial networks (Goodfellow et al., 2016). This convergence of diverse approaches facilitates increasingly nuanced modeling of complex sequence-activity relationships in CRISPR-Cas9 systems, thus ameliorating prediction accuracy.

Secondly, it is notable that studies are progressively endeavoring to augment the practicality of their models by extending their predictive capabilities to diverse scenarios. These encompass a wide range of editing events, including but not limited to the knockout of specific enzymes, off-target cleavage site prediction, and base editing outcome anticipation. This trend elucidates the expanding ambit of applicability for deep learning techniques within this domain, potentially enriching their usability and impact in real-world settings.

Moreover, the ambit of organisms and cell types under investigation in these studies is also steadily widening. While initial investigations predominantly focused on more commonly used organisms and cell cultures, more recent studies have shown promising results in more complex organisms like *Xenopus* and zebrafish embryos or even in the specialized contexts of human blood cells. This broadening scope of research lends credence to the universality and adaptability of deep learning methods in CRISPR-Cas9 editing outcome prediction.

Finally, there is an emergent focus on the interpretability and explicability of models, evident in the incorporation of attention mechanisms and comprehensive evaluation of Cas9 variants. These aspects, although often overlooked in a frenzy to achieve higher accuracy, are paramount to the practical adoption of these models, as they provide insights into the model’s decision-making process and thus increase trust in their predictions.

The emerging field of applying deep learning methods to predict CRISPR-Cas9 editing outcomes is witnessing rapid evolution. The current trends underscore a concerted move towards more sophisticated, practical, and interpretable deep learning solutions, rendering this an exciting and promising research Frontier.

## 5 Deep learning for design of high-activity gRNAs

Accompanying the escalating interest in harnessing the potential of CRISPR-Cas systems for genome editing has emerged a critical need for the design of high-activity guide RNAs (gRNAs). A high-activity gRNA, often fundamental to the successful manipulation of genetic sequences, is characterized by its potent ability to direct the Cas proteins to the intended genomic loci with high efficiency and specificity, thereby maximizing on-target activity and minimizing off-target effects (Mohr et al., 2016). However, the formulation of a high-activity gRNA presents a substantial challenge, as it requires a comprehensive understanding of the complex sequence-activity relationships inherent to gRNAs. The application of deep learning, a sophisticated breed of machine learning, to this conundrum has introduced a promising avenue of exploration.

In the intricate domain of deep learning, algorithms built upon layered architectures of artificial neurons, or nodes, are furnished with the capacity to learn intricate, nonlinear relationships from copious data (LeCun et al., 2015). In the context of high-activity gRNA design, these deep learning architectures are fed with data encompassing gRNA sequences and their associated experimental activities, subsequently learning to extrapolate the underlying relationships that govern gRNA efficiency. This predictive capability of deep learning models effectively translates into a powerful tool for high-activity gRNA design. Table 5 encapsulates a snapshot of recent studies.

**TABLE 5:** Summary of the Contributions of Deep Learning Studies for High-Activity gRNA Design.

Author	Contributions
Baisya et al. (2022)	Presented DeepGuide, a neural network-based architecture for designing high-activity Cas9 and Cas12a sgRNAs, significantly increasing the success rate of CRISPR-based mutagenesis in the yeast Yarrowia lipolytica
Wang et al. (2019)	Introduced an optimized gRNA design for two high-fidelity SpCas9 variants using deep learning. Developed DeepHF, a model combining RNN with essential biological features for activity prediction, outperforming other models and design tools
Feng et al. (2021)	Conducted an in-depth investigation into the effects of guide-target mismatches on dCas9-sgRNA binding activity in bacteria, improving the predictive accuracy of sgRNA design using a biophysical model and a convolutional neural network
Kim et al. (2020a)	Performed an extensive comparison of PAM-sequence compatibilities and the on-target and off-target activities of SpCas9 and its variants, xCas9 and SpCas9-NG, at endogenous sites in human cells. Created deep-learning models to predict the activities of xCas9 and SpCas9-NG.

One pertinent example is DeepGuide (Baisya et al., 2022), a neural network architecture that proved proficient at designing high-activity sgRNAs for Cas9 and Cas12a. The model’s training on genome-wide CRISPR activity profiles significantly bolstered the success rate of CRISPR-based mutagenesis in Yarrowia lipolytica.

In a similar manner, Wang et al. (2019) offered an optimized gRNA design for two high-fidelity SpCas9 variants using a deep learning model known as DeepHF. This model amalgamates Recurrent Neural Network (RNN) with biological features essential for activity prediction, thereby outperforming previous models and design tools.

Investigating guide-target mismatches and their impact on dCas9-sgRNA binding activity, Feng et al. (2021) employed a convolutional neural network to construct a predictive model. This model assists in the rational design of sgRNA in bacterial CRISPR interference. By marrying a biophysical model with deep learning, they significantly improved the predictive accuracy of sgRNA design, demonstrating the efficacy of deep learning in CRISPR system applications.

Further extending the analysis to PAM-sequence compatibilities and the on-target and off-target activities of SpCas9 and its variants, Kim H. K. et al. (2020) performed a comprehensive comparison at endogenous sites in human cells. The introduction of new non-NGG PAM sequences and the creation of deep-learning models for predicting xCas9 and SpCas9-NG activities underscored the potential for these techniques to facilitate genome editing applications.

### 5.1 Remarks on recent research trends

The emergence of deep learning has irrevocably revolutionized the landscape of CRISPR-Cas9 system-based research. The drive to ascertain high-activity gRNAs to increase the efficacy of genome editing tasks has seen the advent of increasingly sophisticated computational tools harnessing deep learning. These tools exhibit an ability to fathom the intricate sequence-activity relationships of gRNAs that have hitherto been elusive.

A confluence of varied deep-learning architectures has been seen in recent studies. From CNNs to RNNs, these methodologies imbibe essential biological features in their models to aid in the accurate prediction of gRNA activities. This incorporation of biological knowledge into the neural network models has undoubtedly improved their performance, outstripping other existing models and design tools.

An innovative trend is the modeling of guide-target mismatches to enhance the accuracy of sgRNA design (Chuai et al., 2018). Leveraging the innate strengths of deep learning, these models are capable of predicting the effects of mismatches on dCas9-sgRNA binding activity, thereby informing the design of sgRNAs.

The deep learning models employed in these studies not only serve to predict but also provide a comprehensive comparison of on-target and off-target activities of SpCas9 and its variants. This permits an evaluation of PAM-sequence compatibilities at endogenous sites in human cells, underscoring the potential for precision in genome editing (Sternberg et al., 2014). As evidenced in these studies, integrating deep learning into the CRISPR-Cas9 systems is leading to transformative progress in the design of high-activity gRNAs. It is anticipated that this trend will continue to evolve, with deep learning models becoming even more adept at handling the challenges inherent in the field of genome editing (Doench et al., 2016).

## 6 Deep learning for automated system implementation in CRISPR-Cas systems

In the era where biological research is progressively influenced by computational methods, the implementation of deep learning-based automated systems has witnessed a progressing interest, especially in the domain of CRISPR-Cas systems. The inception of such systems emanates from the necessity to address the inefficiencies associated with manual handling of the intricacies involved in gRNA design and implementation. Automated systems, empowered by deep learning methodologies, aim to streamline these processes, bringing about a paradigm shift in the execution of CRISPR-Cas system-based experiments.

Deep learning algorithms, with their unparalleled proficiency in understanding and modelling complex patterns, form the bedrock of these automated systems (LeCun et al., 2015). Imbued with the capacity to harness the wealth of experimental and theoretical data, these algorithms are capable of processing numerous variables simultaneously, from gRNA sequence characteristics to environmental factors affecting gRNA performance. The consequent predictive models expedite the decision-making process, underpinning the selection of optimal gRNA designs and their implementation strategies (Cong et al., 2013).

As a manifestation of this approach, recent studies have unveiled automated systems that offer end-to-end solutions, from the design of high-activity gRNAs to their implementation in genome editing tasks (Brinkman et al., 2018). These systems draw upon the prowess of deep learning models, automating not only the gRNA design but also the prediction of potential off-target effects and the evaluation of experimental results. As such, deep learning has contributed significantly to the refinement of CRISPR-Cas systems, rendering them more accessible, precise, and efficient (Doench et al., 2016). The advent of such transformative methodologies has opened the floodgates to explore new dimensions in genome editing, thereby catalyzing the transition towards a more automated and effective use of CRISPR-Cas systems. Table 6 provides an overview of contemporary research endeavors.

**TABLE 6:** Summary of the contributions of deep learning studies for automated system implementation in CRISPR-Cas systems.

Author	Contributions
Cordero-Maldonado et al. (2019)	Utilized a deep learning software based on the open-source Inception v3 library for automated injections in zebrafish embryos. The system enabled high throughput genome editing by injecting CRISPR-Cas9 and DNA constructs as efficiently as an experienced experimentalist
Allen et al. (2022)	Introduced a flow-based imaging platform employing deep learning to study the DNA damage response in human hematopoietic stem and progenitor cells treated with CRISPR-Cas9 and recombinant adeno-associated virus. This system simplified the characterization and screening process of genome-editing parameters
Patino et al. (2021)	Developed an automated single-cell electroporation system integrating deep learning and computer vision strategies for gene editing tasks. They demonstrated its potential in high-throughput, precise cell manipulation applications by delivering gRNA complexes into an induced pluripotent stem cell (iPSC) line
Kanfer et al. (2021)	Proposed a novel pooled screening approach, AI-Photoswitchable Screening (AI-PS), which integrates convolutional neural networks with CRISPRi screening for subcellular phenotyping. Their proof-of-concept screen accurately identified essential factors mediating TFEB relocation

The incorporation of deep learning in the automation of various procedures within the CRISPR-Cas systems presents another area of research worth highlighting. The research by Cordero-Maldonado et al. (2019), for instance, demonstrates the use of a deep learning software grounded on the open-source Inception-v3 library for automating injections in zebrafish embryos. With an impressive accuracy, the system managed to identify the injection site and inject CRISPR-Cas9 and DNA constructs as effectively as a seasoned experimentalist would, thus underscoring its potential in facilitating high-throughput genome editing.

An interesting approach was presented by Allen et al. (2022) through the introduction of a flow-based imaging platform that employs deep learning for studying the DNA damage response in human hematopoietic stem and progenitor cells treated with CRISPR-Cas9 and recombinant adeno-associated virus. Their research highlighted that guide RNAs with a higher genome-editing activity are associated with a more significant DNA damage response, thus simplifying the characterization and screening processes of genome-editing parameters.

The automated systems within CRISPR-Cas research also extends to the development of platforms for high-throughput and precise cell manipulation applications. One such example is the work of Patino et al. (2021), who established an automated single-cell electroporation system that amalgamates deep learning and computer vision strategies for gene editing tasks. Their use of a fully convolutional network (FCN) for accurate nuclei and cytosol location in various cell types proved highly effective, demonstrating the viability of their platform by delivering gRNA complexes into an induced pluripotent stem cell (iPSC) line.

Lastly, Kanfer et al. (2021) proposed AI-Photoswitchable Screening (AI-PS), an innovative pooled screening approach. This approach marries convolutional neural networks with CRISPRi screening for subcellular phenotyping. Their method, which involves training machine learning models on subcellular phenotypes and isolating cells of interest for sequencing through photoactivation, holds significant promise for genome-wide applications, as evidenced by their successful identification of essential factors that mediate TFEB relocation.

### 6.1 Remarks on recent research trends

Leveraging the capabilities of deep learning algorithms not only accelerates the genome editing process but also amplifies its precision and efficiency. From automated injections to single-cell electroporation systems, a clear trend of integrating deep learning with established biological procedures is discernible. The capabilities of deep learning to automate these procedures is a testament to its versatility and power, extending beyond high-activity gRNA design into a broader context of automating procedures within the CRISPR-Cas system.

Deep learning’s prowess in image recognition and processing has been pivotal in enabling automated system implementation. The precise localization of target sites within organisms or cells, identification of various cellular structures, and efficient image-based screening have been made possible and more efficient through the use of deep learning algorithms. In particular, CNNs, known for their excellent performance in image-related tasks, have become a mainstay in most works revolving around image analysis for CRISPR-Cas systems.

Given these innovative implementations and the upward trajectory in applying deep learning within the CRISPR-Cas system, it can be postulated that the Frontier of genome editing is rapidly expanding. These advancements contribute to the broader scientific understanding of deep learning’s capabilities in bioinformatics, subsequently opening doors for further exploration and innovative applications within genome editing. This trend of increasing reliance on deep learning underscores its potential to drive forward the future of genome editing and our understanding of biological systems at large.

## 7 Deep learning for other emerging topics in CRISPR-Cas systems

As the confluence of deep learning and CRISPR-Cas systems continue to shape a new Frontier in the field of genome editing, novel research avenues are persistently emerging. Amidst the traditional applications in high-activity gRNA design and automation of CRISPR-Cas systems, there is a growing application of deep learning algorithms in diverse domains linked to the CRISPR-Cas system, extending the dimensions of its influence.

One remarkable illustration of these emerging topics is the utilization of CRISPR-Cas systems in tandem with deep learning for nucleic acid detection related to specific diseases (Chen et al., 2018). This novel approach has displayed commendable potential in the diagnosis of diseases, identifying nucleic acid sequences characteristic to pathogens or genetic disorders with enhanced accuracy and speed. In a similar vein, deep learning algorithms are leveraged to identify anti-CRISPR proteins, augmenting our understanding of CRISPR-Cas systems’ defense mechanism. Furthermore, the prediction of activities and specificities of different Cas9 variants using deep learning models contributes to the advancement of the CRISPR-Cas toolbox, thereby facilitating more versatile and precise genome editing experiments (Brinkman et al., 2018). Table 7 showcases a collection of recent studies.

**TABLE 7:** Summary of the contributions of deep learning studies for emerging topics in CRISPR-Cas systems.

Author	Contributions
Xie et al. (2022)	Established a colorimetric detection method (RAVI-CRISPR) for nucleic acids using CRISPR-Cas12a and deep learning
Kang et al. (2022)	Utilized CRISPR-Cas system with deep learning for direct histone deacetylase activity detection
Ding et al. (2023)	Proposed a CRISPR-Cas12a/Cas13a bioassay with deep learning for synchronous detection of exosomal proteins in cancer diagnosis
Wandera et al. (2022)	Developed a deep learning model for identifying anti-CRISPR proteins (Acrs)
Upmeier zu Belzen et al. (2019)	Demonstrated the use of deep neural networks for predicting the impact of point mutations on the activity of CRISPR-Cas9 and anti-CRISPR proteins
Park et al. (2022)	Utilized a deep learning-based protein structure prediction approach (AlphaFold2) for protein drug design with Anti-CRISPR proteins
Kim et al. (2020b)	Proposed a deep learning-based computational approach for predicting the sequence-specific activity of 13 SpCas9 variants
Zhang et al. (2021b)	Utilized a deep learning model for evaluating and predicting the editing efficiency, specificity, and PAM compatibility of Cas9 variants
Avsec et al. (2021)	Developed BPNet, a deep learning model for predicting base-resolution chromatin immunoprecipitation binding profiles of transcription factors
Muller et al. (2020)	Employed deep learning to analyze public opinions on CRISPR-Cas9 through Twitter data analysis

Diverse novel applications of deep learning in CRISPR-Cas systems present intriguing research trajectories. For instance, Xie et al. (2022) established a unique colorimetric detection method named RApid VIsual CRISPR (RAVI-CRISPR), incorporating CRISPR-Cas12a and a convolutional neural network. This methodology, synchronized with a smartphone application, was adept in sensitive detection of SARS-CoV-2 and African swine fever virus, illustrating its utility in point-of-care testing.

In the intersection of deep learning, CRISPR-Cas systems, and protein analysis, Kang et al. (2022) devised a method for direct histone deacetylase (HDAC) activity detection. Their research offered a sensitive one-pot assay, exhibiting the potential of such methodologies in precision detection tasks. Similarly, Ding et al. (2023) proposed a CRISPR-Cas12a/Cas13a bioassay coupled with deep learning for synchronous detection of exosomal proteins, opening new vistas for cancer diagnosis.

Advancements have also been made in the detection and understanding of anti-CRISPR proteins (Acrs), as evidenced by Wandera et al. (2022). Their deep learning algorithm unearthed numerous potential Acrs across nearly all CRISPR-Cas types and subtypes. In another protein-focused study, Upmeier zu Belzen et al. (2019) successfully utilized deep neural networks for the functional dissection and engineering of proteins, including ERK, HRas, CRISPR-Cas9, and anti-CRISPR protein AcrIIA4.

Building upon this, Park et al. (2022) introduced a novel approach to protein drug design through accurate structure prediction of Anti-CRISPR proteins using deep learning-based methodologies, presenting new opportunities for countering disease-causing proteins. In line with this, Kim N. et al. (2020) proposed a computational approach to predict the sequence-specific activity of 13 SpCas9 variants, substantiating the potential of deep learning models in gene editing.

Additionally, deep learning has been employed for comprehensive evaluations of the editing efficiency and specificity of several Cas9 variants, as seen in Zhang W. et al. (2021). Their results shed light on optimizing Cas9 variants for precise CRISPR-Cas9 editing. Similar predictive capabilities were showcased by Avsec et al. (2021) through their BPNet model, enabling the discovery of soft syntax rules for cooperative transcription factor binding interactions.

Lastly, Muller et al. (2020) utilized deep learning to gauge public opinions on CRISPR-Cas9, providing a compelling perspective on the societal implications and acceptance of this revolutionary technology. This comprehensive usage of deep learning across diverse aspects of CRISPR-Cas systems encapsulates the immense potential and opportunities it offers for future research.

### 7.1 Remarks on recent research trends

The studies detailed in Table 7 epitomize the increasing ubiquity and indispensability of deep learning methods in the sphere of CRISPR-Cas systems. The manifold utilizations of these techniques to illuminate various domains of CRISPR technology serve as evidence of their escalating prominence. From the inception of colorimetric detection methodologies to the advancement of high-throughput bioassays, these studies are emblematic of an epoch of enhanced efficacy and precision in CRISPR-related procedures. With the confluence of CRISPR and deep learning, previously intractable challenges, such as the identification of anti-CRISPR proteins or the prediction of Cas9 variant activity, now stand within the purview of plausible resolution. Furthermore, deep learning’s contributions extend beyond conventional research spheres, offering novel insights into public sentiment regarding CRISPR-Cas9, as evinced in the Twitter data analysis.

The discernible trends woven into these recent studies underscore an evident shift towards the convergence of biology and computational science. The expanding reliance on deep learning as a tool for disentangling the complexities of CRISPR-Cas systems signifies a transformative phase in scientific exploration. By leveraging the strengths of these computational techniques, researchers are increasingly able to navigate the intricacies of genomic manipulation with unprecedented proficiency. Moreover, the integration of deep learning in these investigations has not only enhanced the accuracy and efficiency of the resultant solutions but also augmented the scope of research, broadening the horizons of discovery. The maturation of this interdisciplinary approach portends a promising future, one where the symbiosis of deep learning and CRISPR-Cas systems may chart a path to novel scientific breakthroughs.

## 8 Conclusion

The synthesis and explication of the knowledge generated from the application of deep learning to the ever-evolving field of CRISPR-Cas systems is a task of cardinal importance. Our collective quest for understanding and leveraging the vast potential of these two groundbreaking domains is a testament to the ceaseless ingenuity of the scientific community. This review, which encompasses a substantial and recent corpus of literature, has undertaken the vital role of not only summarizing but also contextualizing these numerous works of investigation within a broader framework.

Given the rapid evolution of deep learning methodologies and the ceaseless expansion of CRISPR-Cas technology, it is indispensable to continuously scrutinize, distill, and disseminate the amassed knowledge. The deep learning domain has been characterized by an extraordinarily swift pace of development, creating an abundance of innovative methodologies, which has found a fertile ground of application in the expanding arena of CRISPR-Cas systems. This cross-disciplinary integration, marked by remarkable synergy, is continually yielding novel insights that are poised to redefine the boundaries of genetic engineering.

The exploration of the confluence of these two fields, captured in this review, illuminates the transformative potential lying at this intersection. By offering an exhaustive account of the strides made in leveraging deep learning for predicting gRNA activity, the review extends a coherent narrative of progress, successes, and persisting challenges. The importance of this timely discourse stems from its ability to not only guide ongoing research but also to shape the future trajectory of this amalgamated field.

In retrospect, the review underscores the necessity of adaptive learning that is sensitive to the swiftly changing landscape of deep learning and CRISPR-Cas systems. Consequently, the urgency to regularly update the state of knowledge in the field cannot be overstated. This endeavor not only facilitates the sharing of insights across the scientific community but also creates a collaborative environment that is conducive to innovation. In doing so, the review plays a pivotal role in fostering a scientific ecosystem that can respond effectively to emerging challenges and capitalize on new opportunities.

However, in the recent studies, it has been observed that almost all studies utilize independent datasets, with each paper often employing a dataset specific to the authors, a common characteristic of CRISPR-Cas studies. Since it is widely categorized the studies in this review, each category often targets distinct predictions. As illustrated in Tables 3–7, even within the same category, unique research objectives are pursued. Consequently, the metrics used vary significantly, ranging from log-likelihood, accuracy, AUC, to Cosine similarity, and sometimes, the aim is to create embeddings. In such a diverse landscape, a numerical comparison using a common dataset is virtually unfeasible.

The lack of unified reference datasets and common metrics in this field presents a significant challenge in comparing the performance of existing methods numerically. This limitation, inherent to the current landscape of CRISPR-Cas studies, has been considered throughout this review and is worth noting for future studies aiming to advance the field.

In conclusion, the comprehensive overview presented in this review provides a vantage point to appreciate the remarkable progress made in integrating deep learning with CRISPR-Cas systems, particularly in predicting gRNA activity. By illuminating the current state of affairs and shedding light on areas of potential growth, the review serves as a critical beacon for researchers venturing into this dynamic interface of artificial intelligence and genetic engineering. Furthermore, by facilitating a rigorous and systematic understanding of the recent advancements in the field, this review contributes significantly to the ongoing endeavor of navigating the uncharted territories in the application of deep learning to CRISPR-Cas systems. The continued pursuit of such interdisciplinary integration promises to unlock transformative potential that can redefine our understanding and manipulation of biological systems.
